# Optimized-SopungSunkiwon, a Herbal Formula, Attenuates A*β* Oligomer-Induced Neurotoxicity in Alzheimer's Disease Models

**DOI:** 10.1155/2017/7601486

**Published:** 2017-11-07

**Authors:** Jin Gyu Choi, Sun Yeou Kim, Jong Woo Kim, Myung Sook Oh

**Affiliations:** ^1^Department of Life and Nanopharmaceutical Sciences, Graduate School, Kyung Hee University, 26 Kyungheedae-ro, Dongdaemun-gu, Seoul 02447, Republic of Korea; ^2^College of Pharmacy, Gachon University, 191 Hambakmoero, Yeonsu-gu, Incheon 21999, Republic of Korea; ^3^Department of Korean Neuropsychiatry, College of Korean Medicine and Institute of Korean Medicine, Kyung Hee University, 26 Kyungheedae-ro, Dongdaemun-gu, Seoul 02447, Republic of Korea; ^4^Department of Oriental Pharmaceutical Science, College of Pharmacy and Kyung Hee East-West Pharmaceutical Research Institute, Kyung Hee University, 26 Kyungheedae-ro, Dongdaemun-gu, Seoul 02447, Republic of Korea

## Abstract

Alzheimer's disease (AD), the most common form of dementia, is an age-related neurodegenerative disease that is characterized by memory dysfunction, neuronal cell damage, and neuroinflammation. It is believed that AD-related pathology is mostly due to the overproduction of A*β*, especially the oligomeric form (A*β*O), in the brain. Evidence of the effects of multifunctional medicinal herbs in the treatment of AD has been steadily increasing. Optimized-SopungSunkiwon (OSS), a multiherbal formulation that is composed of six medicinal herbs derived from SopungSunkiwon, is a traditional medicine that is prescribed for neurodegenerative disorders in elderly patients. We previously reported that OSS showed an antiamnesic and memory enhancing effect in mice, but it is unknown whether OSS has a protective effect against A*β*O neurotoxicity. In this study, we investigated the effects of OSS in AD models induced by A*β*O* in vitro* and* in vivo*. We found that OSS protected neuronal cells and inhibited the generation of nitric oxide and reactive oxygen species against A*β*O toxicity* in vitro*. These results were confirmed by* in vivo* data that oral administration of OSS for 14 days attenuated memory impairments and neuronal cell death by modulating gliosis, glutathione depletion, and synaptic damage in the mouse hippocampus induced by A*β*O.

## 1. Introduction

Alzheimer's disease (AD) is characterized by progressive memory and learning disorders coupled with severe neuronal degeneration [1]. Although the exact mechanisms of AD pathogenesis remain to be established, it is widely known that amyloid-*β* (A*β*) deposits play a key role in the disease [2]. Among the different forms of A*β*, the oligomeric form (A*β*O) is thought to be primarily related to the pathogenesis of AD because of its neurotoxicity, which impairs functional synaptic plasticity and induces memory loss by inhibiting hippocampal long-term potentiation (LTP) [3–5]. A*β*O has also been implicated in triggering neuronal cell death by activating glial cells and generating reactive oxygen species (ROS) in AD brains [6–8]. These characteristics of A*β*O indicate the potential that A*β*O-induced experimental models to show various pathological features of AD may be useful.

The paradigm of drug discovery for neurodegenerative diseases is currently diverging from a single-target to a multitarget approach, because the effects of single-target drugs are too limited to allow for effective treatment of complex neurodegenerative diseases such as AD [9]. Recent studies have provided considerable evidence showing that the multimodal effects of several herbal extracts or herbal formulations are highly effective in the treatment of AD [10, 11]. For example, EGb761, a standardized extract of* Ginkgo biloba* leaves, inhibits A*β*-induced ROS accumulation, neuronal damage, and formation of A*β* fibrils [12–14]. B401, a herbal formulation that is famous in traditional Chinese medicine and is widely used to treat brain diseases, attenuates glutamate-induced neuronal cell death in SH-SY5Y cells and cognitive dysfunction in triple transgenic AD mice by reducing AD-related pathological proteins including A*β* and tau [15]. Therefore, traditional herbal medicines, which have multitarget and multipotent effects, are emerging as potential treatments options for AD.

Optimized-SopungSunkiwon (OSS) is traditionally prescribed to treat senile constipation and it has been reported that it also works effectively in hyperglycemia, hyperlipidemia, and diabetic nephropathy [16, 17]. OSS consists of the following six medicinal herbs:* Bombyx mori* L.,* Plantago asiatica* L.,* Rheum palmatum* L.,* Poria cocos* Wolf,* Gardenia jasminoides* Ellis, and* Cuscuta chinensis* Lam. A previous study showed that* Bombycis excrementum*, the herb that is present in the largest proportion in the composition of OSS, protects hippocampal neurons and ameliorates memory impairment in mice in which AD-like pathological features are induced by intrahippocampal injection of A*β*O_1–42_ [18]. Moreover, we previously confirmed that OSS treatment results in memory enhancing activity as well as recovery from scopolamine-induced memory loss via the facilitation of acetylcholine release and regulation of synaptic proteins in mice [19]. However, the effect of OSS against A*β* neurotoxicity is yet to be investigated.

In this study, we examined whether OSS displays neuroprotective effects against cognitive deficits, neuronal cell death, neuroinflammation, and synaptic loss in A*β*O_1–42_-induced AD models* in vitro* and* in vivo*.

## 2. Experimental Procedure

### 2.1. Materials

Roswell Park Memorial Institute (RPMI) medium, Dulbecco's modified Eagle medium (DMEM), fetal bovine serum (FBS), horse serum (HS), and penicillin–streptomycin (P/S) were purchased from Hyclone Laboratories, Inc. (Logan, UT, USA). Rabbit monoclonal antiglial fibrillary acid protein (GFAP) and rat monoclonal anti-CD11b (Mac-1) were purchased from Millipore Bioscience Research (Bedford, MA, USA). Rabbit polyclonal antipostsynaptic density protein-95 (PSD-95) was purchased from Abcam (Cambridge, MA, USA). Biotinylated goat anti-rabbit, rabbit anti-rat, goat anti-mouse antibody, normal goat serum (NGS), normal rabbit serum (NRS), and avidin–biotin complex (ABC) kit were purchased from Vector Lab (Burlingame, CA, USA). 3-(4,5-Dimethylthiazol-2-yl)2,5-diphenyltetrazolium bromide (MTT), 2,2-diphenyl-2-picrylhydrazyl (DPPH), 2,2-azinobis-(3-ethyl-benzothiazoline-6-sulphonic acid) (ABTS), 2,7-dichlorodihydrofluorescein diacetate (DCFH-DA), cresyl violet, paraformaldehyde (PFA), 3,3-diaminobenzidine (DAB), sodium chloride, sodium nitrite, collagen, Griess reagent, phosphate-buffered saline (PBS), 1,1,1,3,3,3-hexafluoro-2-propanol (HFIP), dimethyl sulfoxide (DMSO), DMSO anhydrous, and mouse monoclonal antisynaptophysin (SYN) were purchased from Sigma–Aldrich (St. Louis, MO, USA). A*β*_1–42_ peptide was purchased from American Peptide (Sunnyvale, CA, USA). Bradford protein assay was purchased from Bio-Rad Laboratories (Hercules, CA, USA).

### 2.2. Preparation of A*β*O_1–42_ Solution

Soluble oligomers were generated by previously described methods with slight modifications [18]. Briefly, A*β*_1–42_ peptide was dissolved in HFIP to the final concentration of 1 mg/ml at room temperature for 3 days. The peptide was aliquoted and dried under vacuum for 1 h. The aliquoted peptide was dissolved in DMSO anhydrous form to the final concentration of 1 mM. Protein determination was performed by Bradford assay to calculate molarities of solution. The A*β*_1–42_ stock in DMSO anhydrous form was diluted directly into sterilized PBS at 10 *μ*M and incubated at 4°C for 24 h to make oligomeric form of A*β*_1–42_.

### 2.3. Preparation of OSS Extract

OSS was prepared as has been previously described [19]. Briefly, OSS was made from a mixture of the following six herbs:* Bombycis excrementum, Plantaginis Semen, Rhei Rhizoma, Gardenia Fructus, Poria*, and* Cuscutae Semen* (1.5 : 1.5 : 0.5 : 1 : 1 : 1) obtained from the Kyongdong local market (Seoul, Korea). Each herb mixture (400 g) was extracted three times with sonication in distilled water for 2 h. Following filtration, the solution was evaporated in a vacuum and lyophilized (yield: 1.925%). The powder was kept at 4°C before use. This extract was previously standardized by analysis of sennoside A, crocin, and geniposide contents [19].

### 2.4. DPPH Radical Scavenging Activity Assay

Various concentrations of OSS were mixed with 0.20 mM DPPH ethanolic solution (1 : 1). After incubation at dark room temperature for 30 min, the mixture determined at the absorbance of 517 nm using spectrophotometer. Also, the antioxidant activity of OSS was expressed as half maximal inhibiting concentration (IC_50_) which is defined as the concentration of OSS required to scavenge 50% of DPPH radicals. IC_50_ values were estimated by a nonlinear regression. DPPH radical scavenging activity (%) = {control – (sample – blank)}/control × 100.

### 2.5. ABTS Cation Scavenging Activity Assay

7.40 mM ABTS solution was added to 2.60 mM potassium phosphate 1 day before starting the experiment in the dark. Various concentrations of OSS were mixed with 7.40 mM ABTS solution and 2.60 mM potassium phosphate. After incubation at room temperature for 5 min, the mixture determined at the absorbance of 732 nm using spectrophotometer. Also, the antioxidant activity of OSS was expressed as IC_50_, which were estimated by a nonlinear regression. ABTS  cation  scavenging  activity  (%) = (control − sample)/control × 100.

### 2.6. Cell Culture and Treatment

Rat pheochromocytoma PC12 cells were maintained in RPMI, supplemented with 5% heat-inactivated FBS, 10% HS, and 1% P/S in an atmosphere of 5% CO_2_ at 37°C. Mouse BV-2 microglial cells were maintained in DMEM, supplemented with 10% heat-inactivated FBS and 1% P/S in the same conditions. All experiments were carried out 12 h after PC12 and BV-2 cells were seeded in 96-well plates at a density of 2.0 × 10^5^ cells/ml. After the cells were about 70% confluent, various concentrations (0.1–100 *μ*g/ml) of OSS in FBS free media were added to the cells for 24 h at 37°C, with or without 1 *μ*M A*β*O_1–42_. An equal volume of vehicles was administered to the control and toxin groups, for each.

### 2.7. Measurement of Cell Viability

PC12 cells were seeded on 96-well plates and were treated with OSS at doses of 0.1–100 *μ*g/ml for 24 h or pretreated with OSS for 1 h. They were then stimulated with 1 *μ*M A*β*O_1–42_ for 23 additional hours (pretreatment) or 1 *μ*M A*β*O_1–42_ was added for 1 h before treatment with OSS for 23 additional hours (posttreatment). After the treatment, supernatants were removed, and 1 mg/ml of tetrazolium dye (MTT) was added to the cells for 3 h. MTT medium was carefully removed from the wells, and the MTT formazan dye was eluted using dimethyl sulfoxide (DMSO). Absorbance was measured at a wavelength of 570 nm using a spectrophotometer (Versamax microplate reader, Molecular Device; Sunnyvale, CA, USA). Data were expressed as percentages of the values obtained for the controls.

### 2.8. Measurement of Extracellular NO

The accumulated level of NO in culture supernatants was measured using a colorimetric reaction with Griess reagent using a slightly modified variant of the methods that have previously been described [20]. The supernatants (100 *μ*l) were transferred to a separate plate and added to 100 *μ*l of Griess reagent in the dark for 10 min at room temperature. Absorbance at 550 nm was measured. For each experiment, freshly prepared sodium nitrite that had been serially diluted was used as a standard, in parallel with culture supernatants.

### 2.9. Measurement of Intracellular ROS

Intracellular ROS generation was measured with DCFH-DA fluorescence dye, using a slightly modified version of previously described methods [20]. DCFH-DA enters cells passively and is converted into nonfluorescent DCFH, which reacts with ROS to form the fluorescent product dichlorofluorescin (DCF). Cells were seeded onto coverslips in 24-well plates and treated with OSS at 0.1, 1, and 10 *μ*g/mL for 1 h. Then, they were stimulated with 1 *μ*M A*β*O_1–42_ and incubated for an additional 30 min. The cells were incubated with 25 *μ*M DCFH-DA for 30 min. The fluorescence intensity was determined at 485 nm excitation and 535 nm emission, using a fluorescence microplate reader (SpectraMax Gemini EM; Molecular Device, Sunnyvale, CA, USA). Representative images were obtained using a fluorescence microscope (Olympus Microscope System BX51; Olympus, Tokyo, Japan).

### 2.10. Measurement of Total Glutathione

The levels of total glutathione (GSH) were measured using the Total Glutathione Quantification kit (Dojindo Molecular Tech., Tokyo, Japan) according to the instruction manual and previously described method [21]. Briefly, hippocampal tissues were lysed and treated with 5% 5-sulfosalicylic acid. A coenzyme working solution, buffer solution, and enzyme working solution were added to each well at 37°C for 5 min. Then, a GSH standard solution, sample solution, and substrate working solution were added for 10 min each. Absorbance was measured using a spectrophotometer at a wavelength of 405 nm, and concentrations of GSH were determined in the sample solution using a GSH standard curve.

### 2.11. Animals and Surgery Procedure

Male ICR mice (8 weeks, 27–30 g) were purchased from Daehan Biolink Co. Ltd. (Eumseong, Korea). Animals were housed in cages of 5 or 6, had free access to water and food, and were maintained under a constant temperature (23 ± 1°C), humidity (60 ± 10%), and a 12 h light/dark cycle. Animal treatment and maintenance were carried out in accordance with the Principle of Laboratory Animal Care (NIH publication number 85-23, revised 1985) and the Animal Care and Use Guidelines of Kyung Hee University, Seoul, Korea. Stereotaxic injections of A*β*O_1–42_ into mouse hippocampi were performed as previously described [18, 22]. In brief, mice were anesthetized and mounted in a stereotaxic apparatus (myNeuroLab, St. Louis, MO, USA). Each mouse was unilaterally injected (at a rate of 0.5 *μ*l/min) with 3 *μ*l of A*β*O_1–42_ (10 *μ*M) into the granule cell layer (GCL) of the hippocampus (coordinates with respect to bregma in mm: AP −2.0, ML 1.5, DV 2.0), according to a stereotaxic atlas of the mouse brain [23]. Sham-operated mice were injected with the same volume of saline alone. The accuracy of stereotaxic injection to the targeted region was monitored in all animals by examination of the needle tract within brain sections.

### 2.12. Drug Administration

Mice were randomly divided into 5 groups (*n* = 8 in each group), (1) sham group (sham-operated and saline-treated), (2) A*β*O_1–42_ group (A*β*O_1–42_-lesioned and saline-treated), (3) A*β*O_1–42_ + OSS 50 mg/kg/day group (A*β*O_1–42_-lesioned and OSS-treated: 50 mg/kg/day), (4) A*β*O_1–42_ + OSS 100 mg/kg/day group (A*β*O_1–42_-lesioned and OSS-treated: 100 mg/kg/day), and (5) A*β*O_1–42_ + OSS 200 mg/kg/day group (A*β*O_1–42_-lesioned and OSS-treated: 200 mg/kg/day). In all groups, saline and OSS solutions were administered intraorally. OSS dissolved in saline was administered once per day for 14 days (5 days before surgery and for 9 days after surgery).

### 2.13. Step-through Passive Avoidance Test

The step-through passive avoidance test (PAT) was performed according to a method described previously [18]. A learning and memory test was performed using a two-compartment step-through passive avoidance test apparatus. The box was divided into bright and dark compartments (21 × 21 × 21 cm^3^ each) by a guillotine door. The bright compartment contained a 50 W electric lamp, and the floor of the dark compartment was composed of 2 mm stainless steel rods spaced 1 cm apart. Mice were treated with either OSS or vehicle 1 h before the acquisition trial and were initially placed in the bright compartment for the acquisition trial. The door between the two compartments was opened 10 s later. When the hind legs of the mice entered the dark chamber, the guillotine door was closed and an electrical foot shock (0.6 mA) was delivered through the grid floor for 3 s. The mice were again placed in the bright chamber for the retention trial, which was conducted 24 h after the acquisition trial. The time taken for a mouse to enter the dark chamber after the door was opened was defined as the latency time. This was recorded for latencies of up to 300 s.

### 2.14. Novel Object Recognition Test

The novel object recognition test (NORT) was performed according to a method described previously [18]. The experiments were carried out in a grey open field box (45 × 45 × 50 cm^3^). Prior to the test, mice were habituated to the test box for 5 min without the presence of objects. After the habituation period, mice were placed into the test box containing two identical objects and were allowed to explore for 3 min. The objects used in this study were wooden blocks of the same size but different shape. The time spent by the animal exploring each object was measured (defined as the training session). Twenty-four hours after the training session, mice were allowed to explore the objects in the test box for 3 min, during which the familiar object used in the previous training session was placed with a novel object. The time that the animals spent exploring the novel and the familiar objects was recorded (defined as the test session). Animals were considered to be exploring an object when they were facing, sniffing, or biting it. The test box and objects were cleaned with 70% ethanol between sessions. Results were expressed as percentages of novel object recognition time (time percentage = exploring time for novel object/[exploring time for novel object + exploring time for familiar object] × 100).

### 2.15. Brain Tissue Preparation

At 24 h after the memory examination, hippocampal tissue was dissected from the brains of 3 mice from each group in order to measure total glutathione levels. The remaining mice were transcardially perfused with 0.05 M phosphate-buffered saline (PBS) and then fixed with cold 4% PFA in 0.1 M phosphate buffer for cresyl violet staining and immunohistochemistry (*n* = 5 per group). The perfused brains were removed (whole) and postfixed overnight at 4°C in 0.1 M phosphate buffer containing 4% PFA. The brains were then immersed in a solution containing 30% sucrose in 0.05 M PBS for cryoprotection. Coronal sections (30 *µ*m) were serially cut using a freezing microtome (Leica, Nussloch, Germany) and stored in cryoprotectant (25% ethylene glycol, 25% glycerol, 0.05 M phosphate buffer) at 4°C until use in immunohistochemistry.

### 2.16. Cresyl Violet Staining and Immunohistochemistry

For histological assessment of cell loss, free floating sections of mice brains were processed for cresyl violet staining and immunohistochemistry as described in the section above, following a method that had previously been used [18]. For cresyl violet staining, the sections were stained with 0.5% cresyl violet, after which they were mounted onto gelatin-coated slides, dehydrated through graded alcohols (70%, 80%, 90%, and 100%), placed in xylene, and coverslipped using histomount medium. For immunohistochemistry, brain sections were briefly rinsed in PBS and treated with 1% hydrogen peroxide for 15 min. The sections were incubated with a rabbit anti-GFAP antibody (1 : 3000 dilution), a rat anti-Mac-1 (1 : 1000 dilution), a mouse anti-SYN (1 : 200 dilution), or a rabbit anti-PSD-95 antibody (1 : 500 dilution) overnight at 4°C in the presence of 0.3% triton x-100 and NGS or NRS. After rinsing in PBS, the sections were then incubated with biotinylated anti-rabbit IgG, anti-rat IgG, or anti-goat IgG (1 : 200 dilution) for 90 min and with ABC (1 : 100 dilution) for 1 h at room temperature. Peroxidase activity was visualized by incubating sections with DAB in 0.05 M tris-buffered saline (pH 7.6). After several rinses with PBS, sections were mounted onto gelatin-coated slides, dehydrated, and coverslipped with histomount medium. The optical densities of cresyl violet, GFAP, Mac-1, SYN, and PSD-95-positive cells in the dentate gyrus (DG) or CA3 region of the hippocampus were analyzed using ImageJ software (Bethesda, MD, USA). The images were taken at a 400x magnification using an optical light microscope (Olympus Microscope System BX51; Olympus, Tokyo, Japan) equipped with a 20x objective lens. Data are presented as percentages of the sham group values obtained.

### 2.17. Statistical Analysis

All statistical parameters were calculated using GraphPad Prism 5.0 software. Values are expressed as the mean ± standard error of the mean (SEM). Results were analyzed by one-way analysis of variance (ANOVA) analysis followed by the Newman-Keuls multiple comparison post hoc test. Differences with a* p* value lower than 0.05 were considered statistically significant.

## 3. Results

### 3.1. Effect of OSS against A*β*O_1–42_-Induced Neurotoxicity* In Vitro*

**Figure 1:**
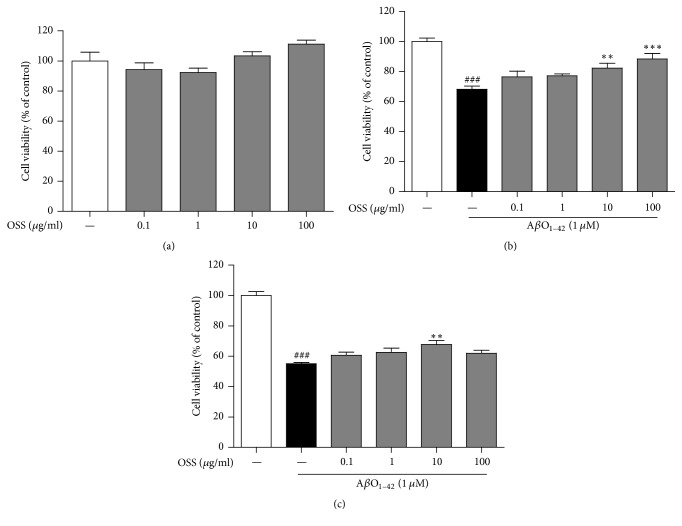
*Neuroprotective effect of OSS on AβO*_1–42_ *toxicity in PC12 cells.* Cells were treated with OSS for 24 h without 1 *μ*M A*β*O_1–42_ (a). The cells were also treated with 1 *μ*M A*β*O_1–42_ 1 h after OSS treatment (b) or 1 h before OSS treatment (c). Cell viability was measured using by MTT assay. OSS treatment alone did not change their viability, while OSS pretreatment or posttreatment protected PC12 cells against A*β*O_1–42_-induced toxicity. Data are expressed as percentages relative to untreated controls. Values are indicated as the mean ± SEM. ^###^*p* < 0.001 compared to the control group; ^*∗∗*^*p* < 0.01 and ^*∗∗∗*^*p* < 0.001 compared to the A*β*O_1–42_-only treated group.

It has been reported that A*β*O_1–42_ induces PC12 cell death by inducing apoptosis [24]. In this study, we investigated whether OSS provides protection against A*β*O_1–42_-induced cell death* in vitro*. Treatment with OSS only at 0.1–100 *μ*g/ml for 24 h showed no significant difference in cell viability compared to the control group (Figure 1(a)). Pretreatment with OSS at 10 and 100 *μ*g/ml significantly inhibited the reduction of cell viability (82.40 ± 3.02% and 88.40 ± 3.60%, resp.) compared with that of the 1 *μ*M A*β*O_1–42_ only treatment group (68.20 ± 2.16%; Figure 1(b)). Posttreatment with OSS at 10 *μ*g/ml also significantly ameliorated cell viability (67.73 ± 2.59%) compared with that of A*β*O_1–42_ only treatment group (55.10 ± 0.96%; Figure 1(c)).

### 3.2. Effect of OSS A*β*O_1–42_-Induced NO Generation* In Vitro*

**Figure 2:**
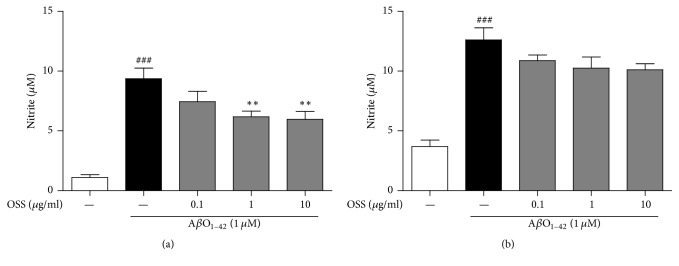
*Inhibitory effect of OSS on AβO*_1–42_ *-induced NO generation in BV-2 microglial cells.* The cells were with 1 *μ*M A*β*O_1–42_ 1 h after OSS treatment (a) or 1 h before OSS treatment (b). NO generation was determined by the nitrite level in the supernatant using the Griess reagent. OSS pre- or posttreatment inhibited overproduction of nitrite level by A*β*O_1–42_ stress. Values are indicated as the mean ± SEM. ^###^*p* < 0.001 compared to the control group; ^*∗∗*^*p* < 0.01 compared to the A*β*O_1–42_-only treated group.

NO plays a key role in a variety of inflammatory statuses, being released in response to pathological stimuli [25]. Excessive concentrations of NO also lead to the formation of oxidative stress cascades, thereby contributing to a neurotoxic cascade such as A*β*-mediated neurodegeneration [26]. To examine the anti-inflammatory effects of OSS against A*β*O_1–42_, we evaluated whether OSS inhibits NO production in activated microglia cells induced by A*β*O_1–42_. Incubation with 1 *μ*M A*β*O_1–42_ increased NO production up to about 10 *μ*M. Compared to the group treated with A*β*O_1–42_ only, the group that underwent pretreatment with OSS at 1 and 10 *μ*g/ml significantly inhibited NO generation (6.18 ± 0.46 *μ*M and 5.98 ± 0.64 *μ*M, resp.) (Figure 2(a)). Posttreatment with OSS showed that NO generation was inhibited compared to the production levels observed in the A*β*O_1–42_ only treatment group. However, the observed difference was not significant (Figure 2(b)).

### 3.3. Antioxidant Effects of OSS* In Vitro* and* In Vivo*

**Figure 3:**
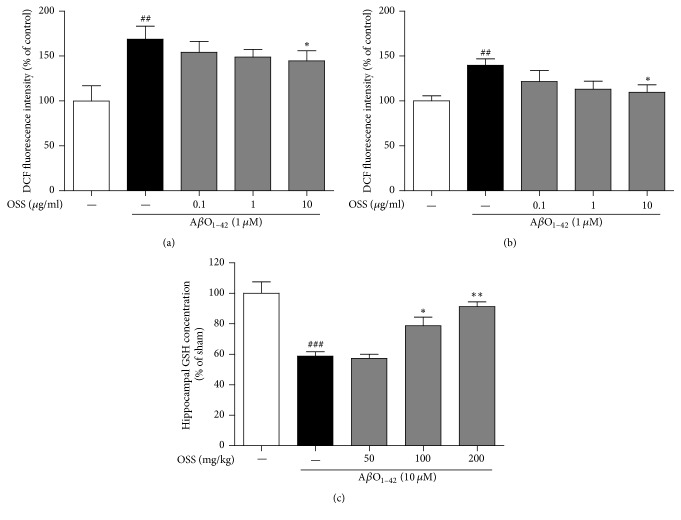
*Inhibitory effect of OSS on AβO*_1–42_ *-induced intracellular ROS generation and hippocampal GSH depletion.* The ROS and GSH levels were measured by the fluorescence dye of DCF and manufactured manual, respectively. Pre- or posttreatment with OSS inhibited ROS generation in PC12 cells (a, b) and reduction of GSH levels in hippocampal tissues (c) against A*β*O_1–42_ toxicity. Data are expressed as percentages relative to untreated controls (intracellular ROS levels) or sham-operated group (hippocampal GSH levels). Values are indicated as the mean ± SEM of four replicates. ^##^*p* < 0.01 and ^###^*p* < 0.001 compared to the control (in PC12 cells) or sham-operated (in hippocampal tissues) group; ^*∗*^*p* < 0.05 and ^*∗∗*^*p* < 0.01 compared to the A*β*O_1–42_-only treated group.

To evaluate the antioxidant potential of OSS, we performed the DPPH free radical and ABTS cation scavenging assay. We found that OSS showed higher scavenging activity than an extract of* Scutellaria baicalensis* Georgi (SBE), used as a positive control in both the DPPH and ABTS assays (Table 1). This trend is in accordance with the inhibitory effects of OSS against A*β*O_1–42_-induced ROS generation. In this study, pretreatment with OSS at 10 *μ*g/ml significantly inhibited ROS generation (144.53 ± 11.44%) when compared to the values obtained with the A*β*O_1–42_ only treatment group (168.77 ± 14.53%; Figure 3(a)). Posttreatment with OSS at 10 *μ*g/ml also led to significantly lower ROS generation values (109.61 ± 8.25%) after A*β*O_1–42_ insult compared to those obtained from the A*β*O_1–42_ only treatment group (139.74 ± 7.20%; Figure 3(b)). Moreover, we investigated the effects of OSS on the induction of GSH as an antioxidant in the mouse hippocampus. The levels of GSH, the most prevalent antioxidant in the brain, consistently decrease with increasing oxidative stress in AD [27, 28]. The hippocampal GSH concentration of 10 *μ*M A*β*O_1–42_-injected vehicle-treated mice was significantly reduced (58.90 ± 2.85%), while treatment with OSS at 100 and 200 mg/kg/day significantly recovered (78.78 ± 5.63% and 91.24 ± 3.23%) GSH concentration. These results indicate that OSS has antioxidant effects against oxidative stress induced by A*β*O_1–42_.

### 3.4. Effect of OSS on Memory Impairment Induced by Intrahippocampal A*β*O_1–42_ Injection in Mice

**Figure 4:**
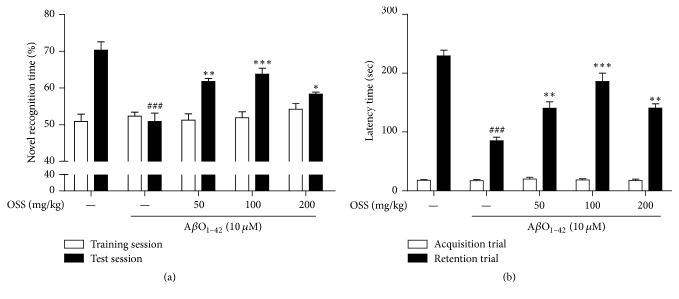
*Effect of OSS on AβO*_1–42_ *-induced memory impairment.* Mice were treated with vehicle or OSS for 14 days, starting from 5 days before stereotaxic injection of A*β*O_1–42_. NORT was carried out at 7 (training session) and 8 (test session) days after A*β*O_1–42_ injection (a). PAT was conducted at 9 (acquisition trial) and 10 (retention trial) days after A*β*O_1–42_ injection (b). The A*β*O_1–42_-injected group treated with OSS exhibited significantly ameliorating memory impairment induced by A*β*O_1–42_ stress. Values are indicated as the mean ± SEM. ^###^*p* < 0.001 compared to sham-operated group; ^*∗*^*p* < 0.05, ^*∗∗*^*p* < 0.01, and ^*∗∗∗*^*p* < 0.001 compared to the A*β*O_1–42_-only treated group.

To investigate whether OSS ameliorated memory impairment in mice receiving an intrahippocampal injection of A*β*O_1–42_, NORT and PAT were performed in this study. In the NORT, the A*β*O_1–42_-injected mice spent similar amounts of time (50.90 ± 2.29%) exploring the novel object and the familiar object during the test session. In contrast, sham-operated mice spent more time exploring the novel object (70.28 ± 2.30%). Treatment with OSS at 50, 100, and 200 mg/kg/day significantly improved A*β*O_1–42_-induced cognitive deficits in this test (61.76 ± 0.81%, 63.75 ± 1.62%, and 58.34 ± 0.60%, resp.; Figure 4(a)). No significant differences in novel object recognition time were found between any of the tested groups during the training session.

In PAT, the mean latency time of the A*β*O_1–42_-injected vehicle-treated group (85.13 ± 6.04 s) was significantly shorter than that of the sham-operated group (229.42 ± 9.72 s). OSS administered at 50, 100, and 200 mg/kg/day significantly reversed the observed effect of the A*β*O_1–42_-injected vehicle-treatment in this test (140.27 ± 11.16 s, 186.08 ± 14.08 s, and 140.51 ± 7.48 s, resp.; Figure 4(b)). No differences in latency time were observed between any of the tested groups during the acquisition trial.

### 3.5. Effect of OSS on A*β*O_1–42_-Triggered Neuronal Atrophy in the Mouse Hippocampus

**Figure 5:**
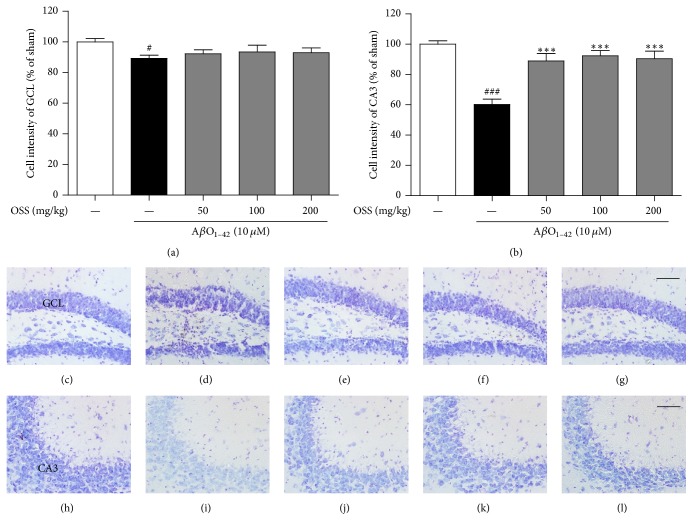
*Effect of OSS on hippocampal cell death induced by AβO*_1–42_ *toxicity. *Mice were treated with vehicle or OSS for 14 days, starting from 5 days before stereotaxic injection of A*β*O_1–42_. Hippocampal cell loss was determined using cresyl violet staining. Quantification was performed by measuring the cell intensity of stained cells in the GCL (a) and in the CA3 (b). Representative photomicrographs are shown for the GCL (c–g) and CA3 (h–l) from each group (400x magnification). Scale bar = 50 *μ*m. (c, h) Sham-operated group; (d, i) A*β*O_1–42_ only treated group; (e, j) A*β*O_1–42_ + OSS 50 mg/kg/day group; (f, k) A*β*O_1–42_ + OSS 100 mg/kg/day group; (g, l) A*β*O_1–42_ + OSS 200 mg/kg/day group. Data are expressed as percentages relative to sham-operated group. Values are indicated as the mean ± SEM. ^#^*p* < 0.05 and ^###^*p* < 0.001 compared to sham-operated group; ^*∗∗∗*^*p* < 0.001 compared to the A*β*O_1–42_-only treated group.

Brain atrophy caused by neuronal death is a pathological hallmark of AD in humans and hippocampal atrophy, in particular, is closely related to memory dysfunction [29, 30]. To further understand the mechanisms underlying the recovery of memory function, the inhibition of A*β*O_1–42_-triggered hippocampal neuronal death by OSS was investigated using cresyl violet staining. The A*β*O_1–42_-induced reductions in neuronal density in the granule cell layer (GCL) of the DG (89.23 ± 1.47%) and CA3 (60.25 ± 2.45%) hippocampal regions were significant compared to those of the sham-operated group. This loss was significantly inhibited by OSS treatment at 50, 100, and 200 mg/kg/day in the CA3 region of the mouse hippocampus (Figure 5).

### 3.6. Effects of OSS on A*β*O_1–42_-Induced Astrocyte and Microglia Activation in the Mouse Hippocampus

**Figure 6:**
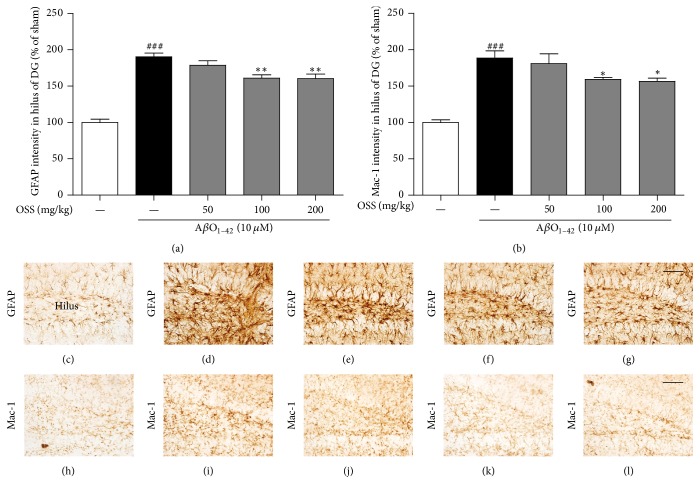
*Effect of OSS on AβO*_1–42_ *-induced hippocampal reactive gliosis.* Mice were treated with vehicle or OSS for 14 days, starting from 5 days before stereotaxic injection of A*β*O_1–42_. Reactive gliosis was determined using GFAP and mac-1 antibody. Quantification was performed by measuring the cell intensity of GFAP-positive (a) and mac-1-positive (b) cells in the hilus region of hippocampus. Representative photomicrographs are shown for the GFAP (c–g) and mac-1 (h–l) stained cells from each group (400x magnification). Scale bar = 50 *μ*m. (c, h) Sham-operated group; (d, i) A*β*O_1–42_ only treated group; (e, j) A*β*O_1–42_ + OSS 50 mg/kg/day group; (f, k) A*β*O_1–42_ + OSS 100 mg/kg/day group; (g, l) A*β*O_1–42_ + OSS 200 mg/kg/day group. Data are expressed as percentages relative to sham-operated group. Values are indicated as the mean ± SEM. ^###^*p* < 0.001 compared to sham-operated group; ^*∗*^*p* < 0.05 and ^*∗∗*^*p* < 0.01 compared to the A*β*O_1–42_-only treated group.

It is known that the activation of astrocyte and microglia under neuroinflammatory conditions plays an important role in the destruction of neurons and leads to synaptic dysfunction, thereby resulting in memory deficits [31]. The intensity of GFAP, a specific marker for astrocytes, in the hilus region of the DG was significantly increased in the A*β*O_1–42_-injected group (190.37 ± 5.10%) as compared with the sham-operated group. This intensity was significantly reduced after OSS treatment at 100 and 200 mg/kg/day (160.89 ± 4.62% and 160.27 ± 6.30%, resp.; Figure 6(a)).

The intensity of mac-1, a specific marker for microglia, in the hilus region of the DG was also nearly doubled in the A*β*O_1–42_-injected group (188.56 ± 9.92%) compared with the sham-operated group. In contrast, mac-1-positive intensity of A*β*O_1–42_-injected mice treated with OSS at 100 and 200 mg/kg/day was significantly decreased (159.25 ± 2.76% and 156.56 ± 4.44%, resp.; Figure 6(b)). Taken together, OSS treatment effectively inhibits hyperactivation of astrocyte and microglia triggered by A*β*O_1–42_ toxicity.

### 3.7. Effects of OSS on Synaptic Damage Derived from A*β*O_1–42_ in the Mouse Hippocampus

**Figure 7:**
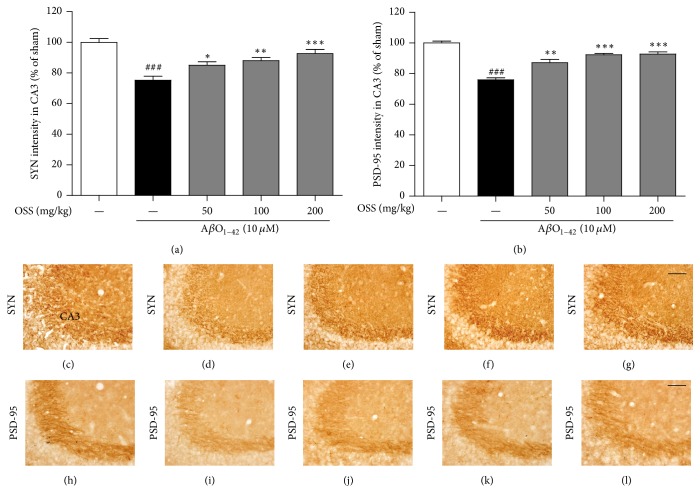
*Effect of OSS on AβO*_1–42_ *-induced presynaptic and postsynaptic damage in the hippocampus.* Mice were treated with vehicle or OSS for 14 days, starting from 5 days before stereotaxic injection of A*β*O_1–42_. The immunoreactivity of SYN (a) and PSD-95 (b) was quantified by measuring the density of each stained area in the hippocampal CA3 region, respectively. Representative photomicrographs are shown for the SYN (c–g) and PSD-95 (h–l) stained cells from each group (400x magnification). Scale bar = 50 *μ*m. (c, h) Sham-operated group; (d, i) A*β*O_1–42_ only treated group; (e, j) A*β*O_1–42_ + OSS 50 mg/kg/day group; (f, k) A*β*O_1–42_ + OSS 100 mg/kg/day group; (g, l) A*β*O_1–42_ + OSS 200 mg/kg/day group. Data are expressed as percentages relative to sham-operated group. Values are indicated as the mean ± SEM. ^###^*p* < 0.001 compared to sham-operated group; ^*∗*^*p* < 0.05, ^*∗∗*^*p* < 0.01, and ^*∗∗∗*^*p* < 0.001 compared to the A*β*O_1–42_-only treated group.

Growing evidence shows that A*β*O induce depletion of hippocampal synaptic proteins such as SYN and PSD-95, resulting in memory dysfunction in AD [32, 33]. As shown in Figure 7, the immunoreactivity of both SYN and PSD-95 in the hippocampal CA3 region was markedly decreased in the A*β*O_1–42_-injected group (75.21 ± 2.66% and 75.95 ± 1.27%, resp.) compared with sham-operated group. This trend was significantly reversed by OSS treatment at 50, 100, and 200 mg/kg/day for both SYN (85.10 ± 2.27%, 88.10 ± 1.96%, and 92.65 ± 2.70%, resp.) and PSD-95 (87.18 ± 2.12%, 92.32 ± 0.93%, and 92.74 ± 1.38%, resp.). These data suggest that OSS restores A*β*O_1–42_-induced synaptic disruption, which is linked to the amelioration of memory impairment.

## 4. Discussion

A*β*O, the most toxic form of A*β*, is considered to play a central role in AD pathogenesis rather than A*β* monomers or fibrils [34, 35]. He et al. demonstrated that memory impairment and hippocampal CA1 neuronal damage were more remarkable in A*β*O_1–42_-infused rats than in those where features of AD pathology were induced by A*β*_1–42_ fibrils due to the observation that A*β*O_1–42_ more evidently exhibited proinflammatory factor stimulation than A*β*_1–42_ fibrils [36]. Our present data shows that systemic treatment with OSS ameliorates memory dysfunction by blocking A*β*O_1–42_-induced hippocampal cell damage, hippocampal GSH depletion, glial hyperactivation, and synaptic disruption in a mouse model of AD. It was also confirmed that OSS directly inhibited A*β*O_1–42_-induced cell degeneration as well as overproduction of NO and ROS* in vitro*.

Oxidative stress is an important pathological factor of AD [37]. Several studies indicate that A*β*_1–42_ peptide is at the center of oxidative damage as it is an indicator of ROS generation in AD brains [38]. Additionally, increased A*β*-mediated ROS generation can damage the endogenous antioxidant GSH and enzymes such as superoxide dismutase, GSH peroxidase, and catalase, thus inducing A*β* deposits to form in the brain [39]. A*β* deposits stimulate activation of nearby microglia and astrocytes, generating an inflammatory response through the release of proinflammatory mediators [40]. It has been suggested that activated glia are involved in neuronal degeneration because they produce potent toxic molecules including NO and cytokines [41, 42]. The present study demonstrates that OSS treatment inhibits ROS generation in PC12 cells and restores GSH contents depleted by A*β*O_1–42_ in hippocampal tissue. Intracellular ROS concentration and endogenous oxidant system normalized by OSS also has an influence on the deactivation of glial cells in the hippocampus as well as on the reduction of NO production in BV-2 microglia cells.

The hippocampal synapse network originates from axons of the CA3 pyramidal region, which connect to almost all regions of the hippocampus [43, 44]. Furthermore, CA3 synapses modulate homeostatic plasticity connected to hippocampal neurons [45]. Thus, a marked decrease of synaptic density in the hippocampal CA3 region is highly relevant to synaptic disruption, which is closely linked to memory decline in the pathogenesis of AD [46]. Soluble A*β*O impair hippocampal LTP and can also induce memory dysfunction [3, 32]. In this study, it has been demonstrated that OSS treatment rescues synaptic damage in the hippocampal CA3 region based on the results obtained using SYN and PSD-95 markers, which are specific pre- and postsynaptic proteins, respectively [47, 48]. In this context, it can be hypothesized that the restoration of memory function after OSS treatment is mediated by facilitated hippocampal synapses.

Other approaches investigating potential therapeutics for AD indicate that the design of multitarget drugs is increasingly necessary because most single-target candidates have been unsuccessful in the treatment of AD given that it is a complex and multifaceted pathogenesis [49, 50]. This paradigm of drug discovery for AD is in accordance with the multifunctional actions of medicinal herbs. The neuroprotective effects of OSS observed in this study can be due to each individual herb of OSS. Water extract from silkworm feces (Bombycis excrementum), for example, was shown to protect hippocampal neurons and memory impairment induced by A*β*O_1–42_ in our previous report [18]. Rhaponticin and rhapontigenin isolated from rhubarb roots (Rhei Rhizoma) significantly inhibit A*β*_1–42_-induced apoptotic mechanisms by regulating Bax/Bcl-2 proapoptotic genes in human neuroblastoma cells [51]. Additionally, the protective effects of Poria water extract against A*β*_1–42_-mediated cell death in PC12 cells were also reported [52]. Furthermore, geniposide, one of the active compounds of* Gardeniae fructus*, has been shown to exhibit multifunctional neuroprotective effects by blocking receptors for advanced end product-mediated signaling in APP/PS1 transgenic mice and BV-2 microglia cells [53, 54]. These constituents of OSS may have contributed to its neuroprotective effects against A*β*O_1–42_ neurotoxicity.

## 5. Conclusion

In summary, OSS treatment alleviates A*β*O_1–42_-induced damage of memory function and hippocampal neurons. This effect is likely to be mediated by the inhibition of oxidative stress, neuroinflammation, and decline in hippocampal synaptic density. Further detailed investigation is required to reveal the underlying mechanisms that might explain how OSS treatment regulates neuroinflammation and hippocampal neuronal and synaptic damage. Taken together, our data suggest that OSS may be a potential multitargeted candidate for AD treatment.

## Figures and Tables

**Table 1:** *IC*_*50*_ *values for DPPH and ABTS radical scavenging activity.* OSS showed higher DPPH free radical and ABTS cation scavenging activity than that of positive control. Data were expressed as IC_50_ values. OSS: Optimized-SopungSunkiwon extract and SBE: *Scutellaria baicalensis* Georgi extract (used as a positive control).

	IC_50_ (*μ*g/mL)	
	DPPH assay	ABTS assay
OSS	18.33	25.34
SBE	27.75	25.40
